# Evaluation and validation of a patient-reported quality-of-life questionnaire for Parkinson’s disease

**DOI:** 10.1186/s41687-022-00427-0

**Published:** 2022-03-02

**Authors:** Pantelis Stathis, George Papadopoulos

**Affiliations:** Department of Neurology, Mediterraneo Hospital, 8-12 Ilias Street, 16675 Glyfada, Athens, Greece

**Keywords:** Quality of life, Parkinson’s disease, Questionnaire, Patient-reported outcomes

## Abstract

**Background:**

Parkinson’s disease (PD) is a chronic, progressive illness with a profound impact on the health-related quality of life (HRQoL). Disease-specific patient-reported HRQoL measures, such as PDQ-39 and its short version PDQ-8, are increasingly used in clinical practice to address the consequences of PD on everyday life. Due to limitations in the content, especially in non-motor symptoms and sleep disturbances of PDQ-8, PDQoL7, a 7-item, short-term, self-reported, PD-specific HRQoL questionnaire was developed.

**Methods:**

A representative sample of 60 adults with idiopathic PD completed the PDQoL7 questionnaire and the existing validated PDQ-8 and EQ-5D-5L questionnaires (all in Greek).

**Results:**

PDQoL7 summary index strongly correlated with PDQ-8 (r_s_ = 0.833, *P* < 0.001) and EQ-5D-5L (r_s_ =  − 0.852, *P* < 0.001). The correlation between PDQoL7 and EQ-5D-5L was statistically significantly stronger compared to PDQ-8 and EQ-5D-5L (r_s_ =  − 0.852 vs rs =  − 0.789 respectively, *P* < 0.001). The internal consistency of PDQoL7 was not affected by item deletion (positive item to total correlations: 0.29–0.63). No redundant items (with inter-item correlation coefficients greater than 0.80) were identified. Cronbach’s α for PDQoL7 was comparable to PDQ-8 (0.804 versus 0.799 respectively). As PDQoL7 had three-dimensional structure, omega coefficient analysis confirmed its reliability (omega total: 0.88; omega hierarchical: 0.58).

**Conclusions:**

PDQoL7 is an acceptable, easy to use, valid and reliable tool for the determination of HRQoL in PD patients that is potentially more comprehensive than PDQ-8 based on the available evidence. PDQoL7 could allow for a more thorough evaluation of the impact of PD and contribute to guiding healthcare decisions. This will be confirmed in subsequent analysis on larger patient cohorts.

**Supplementary Information:**

The online version contains supplementary material available at 10.1186/s41687-022-00427-0.

## Introduction

Health-related quality-of-life (HRQoL) is a broad multidimensional concept that reflects the subjective perceptions of patients on the impact of their disease on physical, mental, emotional, and social functioning and overall well-being [1, 2]. Self-reported HRQoL-tools, both generic and disease-specific, were developed over the last decades and are increasingly used as outcomes in clinical studies of chronic diseases both to quantify the burden of the disease on the patients’ everyday life, the impact of treatment and guide health policy decisions [3, 4]. HRQoL assessments are of clinical value provided that they fulfil certain requirements [5]. These include valid theoretical basis (construct validity), content validity, reproducibility, acceptability by respondents, clarity, cultural validity, inter-relatedness of items to allow for the determination of the same underlying construct (internal consistency), ability to detect changes over time in the measured construct (responsiveness), unidimensionality and in the context of clinical trials practicability and ease of use [5].

Parkinson’s disease (PD) is a chronic, progressive neurodegenerative disease that severely affects the HRQoL of patients compared to healthy controls [6]. The correlates of poor HRQoL in PD patients are multifactorial and include demographics (such as age and gender), PD clinical characteristics (such as severity of motor and non-motor symptoms, PD subtypes, disease duration), adverse effects of treatment, comorbid conditions, and psychosocial function [7–9]. Both generic and PD-specific HRQoL tools have been used in PD patients [3, 6, 10–13]. Among the currently available PD-specific HRQoL tools, the Parkinson’s disease questionnaire-39 item (PDQ-39) that contains 39 items that are grouped under 8 domains, and its shorter version PDQ-8 that contains 8 items, each representing a domain of the PDQ-39 are more widely used [10, 14–17]. PDQ-8 can be used as part of the original PDQ-39 questionnaire (nested) or independently [18]. Due to is brevity, PDQ-8 requires less time to complete, but has lower reliability and validity compared to PDQ-39 [12, 19–21]. Despite their broad use, a major limitation of PDQ-39 and PDQ-8 is the lack of clarity in some items that could lead to misconceptions as well as the lack of items on most non-motor symptoms [10, 12]. However, non-motor symptoms can have a detrimental impact on PD prognosis and the overall health status of the patients that exceeds that of motor symptoms [22–28]. Non-motor symptoms, such as night sleep problems, drooling, fatigue, urination problems and dizziness are independent predictors of poor QoL [11, 25, 29]. They are, however, not included in PDQ-8 items. For instance, sleep disorders stand out among non-motor symptoms, given their high prevalence and severe impact on cognitive function and HRQoL [28, 30–33]. Ιncreasing evidence suggests that sleep problems and non-motor symptom burden do not only correlate with HRQoL assessments, but can also be predictive of longitudinal ΗΡQoL change in PD patients [6, 23, 24, 34–37]. In addition, poor sleep quality in PD is associated with depression, anxiety and advanced disease [36]. Another disadvantage of the PDQ-8 tool is that the patient should answer every question recalling the last month’s events, impressions, feelings and thoughts, something not so easy taking also in account the possible cognitive impairment of the PD patient.

Thus, the need for a convenient, reliable, with short recall period, easy to answer, clear and comprehensive tool which covers the range of symptoms of PD patients could contribute towards a better HRQoL estimation. The objective of this study is the development of a content-valid PD-specific HRQoL questionnaire, PDQoL7, that maintains some of the features of PDQ-8, such as the ease of use, and contains improvements in item content to assess the impact of PD on physical, mental and social aspects.

## Materials and methods

### PDQoL7 development

PDQoL7 is a 7-item self-reported PD-specific HRQoL questionnaire that relies on the patient’s perception of their well-being during last week, in 7 discrete domains (one item per domain). The selection of the items that were included in PDQoL7 was based on consultation with healthcare professionals that have experience in PD patients, symptoms spontaneously reported by PD patients during routine visits to healthcare professionals (without being actively asked by healthcare professionals), as well as problems encountered by PD patients due to ambiguity in content or inability to understand some items in the existing PDQ-39/PDQ-8 tools [38]. For example, regarding question 7 of PDQ-8 *“Had painful muscle cramps or spasms”,* patient’s answer should focus only on the pain-accompanied muscle tone alterations (i.e., painful early morning dystonia), omitting other very common painful conditions that could exist in PD [25, 26]. The selected items that were included in PDQoL7 were also confirmed by literature review [8, 9, 11, 12, 19].

PDQoL7 was developed in Greek language. It has 7 items, each corresponding to a distinct domain with 4 possible scores (0 = never, 1 = rarely, 2 = sometimes; 3 = often, 4 = always), and assesses the frequency with which patients experience difficulties due to PD in: (1) Mobility addresses problems of mobility (difficulties with walking, moving the hands or changing position in bed) (2) Skills & Personal care addresses difficulties with activities such as working, driving, hobbies, housework, personal hygiene and getting dressed. (3) Social Life & Communication addresses perceived support from social relationships, for example, feeling isolated or having problems in communication with close relationships, family, or friends, (4) Problems from non-motor symptoms addresses the most commonly reported non-motor symptoms associated with PD, such as pain, fatigue, sialorrhea, constipation, frequent urination, orthostatic phenomena, (5) Emotional status addresses emotional problems, such as feeling depressed (6) Mental status addresses difficulties with attention *or having trouble* in maintaining *focus* and (7) Sleep addresses the presence of sleep problems, night-time sleep problems, or drowsiness during the day. For comparison purposes, the questions of PDQoL7 and PDQ-8 are provided in Additional file 1: Fig. 1).

The validity of PDQoL7 was explored by correlating it to the validated Greek versions of PDQ-8 (PDQ-8^Grv^) and EQ-5D-5L [21, 39]. Lower scores for both PDQoL7 and PDQ-8 represented a higher HRQoL, whereas the opposite applies for EQ-5D-5L index score (range 0–1) with higher scores indicating higher health utility. PDQ-8 was chosen for comparison purposes because the number of items and time required for completion are comparable to PDQoL7.

### Study subjects

This study was conducted in the Outpatient Parkinson’s Disease practice of Mediterraneo Hospital (Athens, Greece). The study received approval from the institution’s scientific committee and all patients provided informed consent. Sixty adult idiopathic PD patients, according to the criteria of the International Parkinson and Movement Disorder Society and the UK *Parkinson's Disease* Society Brain Bank that did not suffer from dementia or conditions that would interfere with the study’s assessments were consecutively enrolled from June 2019 to December 2019. During a single visit, medical history and records were reviewed, the patients underwent clinical examination, and were instructed to complete PDQoL7, PDQ-8 and EQ-5D-5L QoL. All three questionnaires were completed on the same day within approximately 5 min each and were reviewed by the rater(s) for response clarity and completion. Other routine assessment tools that were completed were the MDS-UPDRS Greek official version, Part I: item 1.1 for cognitive impairment, and 1.3 for depressed mood; Part IV: items 4.1–4.4 (% off and disability due to off and % levodopa-induced dyskinesia-LID, and disability due to LID), and the non-motor questionnaire (NMSQuest) [13, 40, 41]. The collected variables also included PD staging (modified Hoehn and Yahr scale) and PD subtype (based on motor signs and PD onset) [42, 43]. Wearing-off and LID % scores were combined with weights based on the disability due to off-state and the disability due to LID, to create two indexes: Disability-Off and Disability-LID.

### Statistical analysis

Categorical variables were presented as absolute (N) and relative frequencies (%) and continuous variables are presented as mean values (standard deviation: SD) and/ or median values (Interquartile Range: IQR, expressed as the 25th–75th percentile of their distribution). Floor and ceiling effects were assessed by means of frequencies. Normality of the continuous characteristics’ distribution was tested through the P-P plots and the Shapiro–Wilk test. The criterion validity (convergent validity) of PDQoL7, i.e., the extent to which it correlates with another tool that measures HRQoL in PD patients, was determined versus the Greek validated versions of the PD-specific PDQ-8 and the generic HRQoL EQ-5D-5L using Spearman’s correlation coefficient (r_s_) [21, 39]. EQ-5D-5L QoL questionnaire has been validated in PD patients [44]. The EQ-5D-5L is a 5-item questionnaire that measures a related, but different construct to PDQoL7/PDQ-8 by evaluating mobility, self-care, daily activities, pain/discomfort, as well as anxiety/depression on a 5-level Likert scale (no problems, slight problems, moderate problems, severe problems and extreme problems, ranging from 1 to 5 points) [45].

The internal consistency (reliability) and validity of PDQoL7 and PDQ-8 were assessed by Cronbach’s α coefficient and inter-item correlations. Cronbach’s α value > 0.70 was used for group-level comparisons. For item-total correlation each item had to correlate with the total score with r > 0.2 (Pearson’s correlation). Cronbach’s α in the range of 0.70–0.95 was considered as an adequate measure of internal consistency.

The assumption that underpins the summing of rating scale items into a total score is their unidimensionality, i.e., that the items represent a common underlying construct [5, 14]. Exploratory Factor Analysis (EFA) using the extraction method of Principal Components was applied to identify the different components of both instruments (PDQoL7, PDQ-8). The specific methodology has been chosen, as it constitutes one of the standard and most widely used statistical methodologies for demonstrating the construct validity of QoL questionnaires [46–48]. To ensure suitability for conducting EFA, we used the Kaiser–Mayer–Olkin (KMO) test and the Bartlett’s test of sphericity. The orthogonal rotation (Varimax method) was used to simplify the factors’ structure and to enhance their interpretability. To determine the number of factors to be kept we used the criteria of eigenvalues > 1.0 and the ‘elbow’ of the scree plot, in addition to the Monte Carlo Parallel analysis, which is an alternative technique that compares the scree plots of factors of the observed data with those of a random data matrix of the same size as the original [49–51]. For each factor, component loadings were interpreted using the following cut-offs (non-normal distributions): ≥ 0.5 relevant, ≥ 0.6 good, and ≥ 0.7 very good. It is noted that a sample size of at least 10 observations per item is usually required to obtain reliable high-quality factor analysis results and to avoid computational difficulties, yet as suggested in the literature, a sample size of N = 50 is considered to be a reasonable absolute minimum [52, 53]. For a sample size of 50, a loading of 0.722 was considered significant. Moreover, the omega Hierarchical was also calculated for both scores (PDQoL7 and PDQ-8 scores) so as to estimate their precision in measuring one general/ overall construct.

In addition to the EFA, the hierarchical cluster analysis based on the Ward’s linkage and the squared Euclidean distances was also applied, in order to identify the instruments’ structure and to compare it with components provided by the EFA. Finally, univariable and multivariable linear regression analysis was performed to test the impact of various demographic and clinical characteristics of the patients on the PDQoL7 and PDQ-8 scores. Stepwise multiple regression analysis was used to determine the factors that best accounted for the variance in HRQoL scores.

## Results

### Demographics and clinical characteristics

The basic demographic and clinical characteristics of the study patients are summarized in Additional file 1: Table 1. Patients of male:female ratio 55:45%, with mean age (± SD) 64.52 ± 9.39 years were enrolled and were equally distributed between akinetic-rigid and tremor-dominant subtypes. Most of the patients were of stage 2–3 (modified Hoehn and Yahr scale) in PD severity (73.4%), had normal cognition or up to mild cognitive impairment and were affected with 1–10 nonmotor symptoms (43.3%). Slight or up to moderate depressed mood was reported by 95% of the subjects.

Descriptive statistics of the scores for each item of PDQoL7 and PDQ8 are shown in Additional file 1: Table 2. The mean (± SD) PDQoL7 and PDQ-8 scores were respectively 14.58 ± 5.55 and 11.82 ± 6.04. There were no missing data. Furthermore, there was no evidence of floor or ceiling effects with only 3.3% and 1.7% of the study patients scoring the lowest value in PDQoL7 (score 4) and PDQ-8 (score 0) respectively, while 1.7% of the patients scored the maximum in either instrument [PDQoL7 (score = 24), PDQ-8 (score = 23)].

### Correlation between PDQoL7, PDQ-8 and EQ-5D-5L

PDQoL7 and PDQ-8 were strongly correlated (Spearman’s correlation r = 0.833, *P* < 0.001). Either tool correlated strongly with PD duration since diagnosis, PD severity, NMSQuest score and MDS-UPDRS-assessed cognitive impairment, disability-Off index, and depressed mood (Table 1). In addition, both scores were significantly and negatively associated with the EQ-5D-5L index score, while it should also be noted that the correlation between the PDQoL7 and the EQ-5D-5L index score (r_s_ = − 0.852; 95% CI = [− 0.909, − 0.794]) was significantly stronger (*p* < 0.001), when compared to the one between the PDQ-8 and the EQ-5D-5L index score (rs = − 0.789; 95% CI = [− 0.842, − 0.669]).

**Table 1:** Spearman’s correlation between PDQoL7/PDQ-8 questionnaire scores and demographic/clinical variables

Demographic/clinical parameters	Spearman’s Correlation coefficients	
	PDQoL7	PDQ-8
Age	0.170	0.204
Number of years since diagnosis	0.528**	0.539**
Modified Hoehn & Yahr stage	0.561**	0.552**
*PD subtype*	− 0.034	0.023
Motor signs	0.018	− 0.052
Onset	− 0.145	− 0.098
Cognitive impairment	0.575**	0.570**
Disability-Off index	0.682**	0.669**
Disability-LID index	0.278*	0.334**
Non-motor questionnaire	0.681**	0.540**
Depressed mood	0.650**	0.593**
EQ-5D-5L index score	− 0.852**	− 0.789**

Strong correlations (≥ 0.50) are indicated in bold

*P < 0.05; **P < 0.01

Both for PDQoL7 and PDQ-8, no redundant items (with inter-item correlation coefficients greater than 0.80) were identified (Table 2). PDQoL7 and PDQ-8 also had adequate internal consistency (PDQoL7 total Cronbach’s α: 0.804; intraclass correlation coefficient, 95% CI: 0.716–0.872 and PDQ-8 total Cronbach’s α: 0.799; intraclass correlation coefficient, 95% CI: 0.711–0.868). The internal consistency for either of the two questionnaires was not affected by item deletion and the reliability analysis (positive item to total correlations in the range of 0.29–0.63) was satisfactory.

**Table 2:** Results for reliability analysis for PDQoL7 and PDQ-8

PDQoL7 items	Inter-item correlations							Item-total correlation	Cronbach’s α if item deleted
	Item 1	Item 2	Item 3	Item 4	Item 5	Item 6	Item 7		
*PDQoL7*									
Item 1	–	0.748^**^	0.281^*^	0.530^**^	0.324^*^	0.345^**^	0.163	0.604	0.767
Item 2	0.748^**^	–	0.289^*^	0.467^**^	0.284^*^	0.480^**^	0.310^*^	0.634	0.760
Item 3	0.281^*^	0.289^*^	–	0.452^**^	0.544^**^	0.433^**^	0.086	0.511	0.784
Item 4	0.530^**^	0.467^**^	0.452^**^	–	0.483^**^	0.402^**^	0.072	0.607	0.767
Item 5	0.324^*^	0.284^*^	0.544^**^	0.483^**^	–	0.353^*^	0.299^*^	0.552	0.776
Item 6	0.345^**^	0.480^**^	0.433^**^	0.402^**^	0.353^*^	–	0.279	0.563	0.774
Item 7	0.163	0.310^*^	0.086	0.072	0.299^*^	0.279	–	0.285	0.815

PDQ-8 items	Inter-item correlations								Item-total correlation	Cronbach’s α if item deleted
	Item 1	Item 2	Item 3	Item 4	Item 5	Item 6	Item 7	Item 8		
*PDQ-8*										
Item 1	–	0.716^**^	0.452^**^	0.272^*^	0.489^**^	0.333^*^	0.243	0.381^**^	0.669	0.748
Item 2	0.716^**^	–	0.406^**^	0.297^*^	0.514^**^	0.225	0.375^**^	0.449^**^	0.702	0.742
Item 3	0.452^**^	0.406^**^	–	0.499^**^	0.364^**^	0.165	0.176	0.284^*^	0.521	0.774
Item 4	0.272^*^	0.297^*^	0.499^**^	–	0.202	0.566^**^	− 0.075	0.397^**^	0.455	0.784
Item 5	0.489^**^	0.514^**^	0.364^**^	0.202	–	0.336^**^	0.282^*^	0.265^*^	0.553	0.770
Item 6	0.333^*^	0.225	0.165	0.566^**^	0.336^**^	–	0.047	0.456^**^	0.451	0.785
Item 7	0.243	0.375^**^	0.176	− 0.075	0.282^*^	0.047	–	− 0.003	0.235	0.815
Item 8	0.381^**^	0.449^**^	0.284^*^	0.397^**^	0.265^*^	0.456^**^	− 0.003	–	0.483	0.780

*P < 0.05; **P < 0.01

The degree of agreement (concordance) in responders’ assessments was low both for PDQoL7 and PDQ-8 (PDQoL7: Kendall’s Coefficient of Concordance W = 0.192, *P* < 0.001 and for PDQ-8 W = 0.088, *P* < 0.001).

### Principal component factor analysis/hierarchical cluster analysis

The PDQoL7 items seemed to be related and therefore suitable for structure detection via factor analysis (Bartlett’s test of sphericity χ^2^ = 136.4, *P* < 0.001). Similar results were obtained for PDQ-8 (χ^2^ = 162.1, *P* < 0.001). Three components had eigenvalues greater than 1 accounting collectively for almost 76% of the variance in the PDQoL7 scores (Additional file 1: Table 3). For PDQoL7, component 1 included “Mobility” and “Skills & Personal-care”, component 2 “Social life & Communication” and “Emotional status” and component 3 “Cognition & Sleep”. The omega hierarchical of PDQoL7 was 0.58, while the omega total was 0.88.

For PDQ-8, 2 components accounted for almost 60% of the variance (Additional file 1: Table 4). Component 1 included the items “Mobility”, “Activities of daily living”, “Cognitions”, and “Bodily discomfort”, and component 2 the items “Social support”, “Communication”, and “Stigma”. The item “Emotional well-being” moderately correlated with both components. The omega hierarchical of PDQ-8 tool was 0.51, while the omega total was 0.87.

Hierarchical cluster analysis identified three clusters for PDQoL7 and two clusters for PDQ-8 (Additional file 1: Table 5).

### Regression analysis

Univariate regression models demonstrated that the factors which significantly affected the PDQoL7 score were the number of years since diagnosis, PD severity, cognitive impairment, Disability-Off and Disability-LID indices, NMSQuest score, and depressed mood (*P* < 0.05) (Additional file 1: Table 6). The aforementioned factors also affected PDQ-8. Increases in PD severity or NMQuest score or depressed mood were associated with comparable increase in PDQoL7 or PDQ-8 scores (a change by 1 in these factors would increase PDQoL7 score by 4.22, 4.83 and 4.33 respectively).

The statistically significant factors that had an impact on the PDQoL7 or PDQ-8 scores are shown in Table 3. The proposed models accounted for 61.2% of the variance in the PDQoL7 score with a good fit for the data (ANOVA F-test: 32.08, < 0.001) and 53.6% of the variance in the PDQ-8 score with a good fit for the data (ANOVA F-test: 23.75, *P* < 0.001).

**Table 3:** Stepwise multiple regression analysis for PDQoL7 and PDQ-8 scores

Clinical features	Estimate (95% CI)	Standard error	*P*-value	Adjusted R^2^
*PDQoL7*				
Disability-Off index	0.15 (0.07, 0.23)	0.04	0.001	0.612
Disability-LID index	0.11 (0.02, 0.21)	0.05	0.024	
Non-motor questionnaire score	3.38 (1.98, 4.79)	0.70	< 0.001	
*PDQ-8*				
Disability-Off index	0.18 (0.09, 0.27)	0.05	< 0.001	0.536
Disability-LID index	0.15 (0.03, 0.27)	0.06	0.012	
Cognitive impairment	2.30 (0.85, 3.75)	0.72	0.002	

## Discussion

This study describes the development of the PDQoL7 questionnaire for the assessment of the impact of PD on the HRQoL. This short easy to complete tool was developed mainly to cover limitations in the content of the existing PDQ-8. The validity, reliability and the overall properties of PDQoL7 were determined in accordance with scientific best practices and corresponding quality criteria by combining the gaps in existing widely used HRQoL tools for PD patients with findings from the literature review, expert clinician advice, and patient interviews [38, 54, 55]. Consequently, PDQoL7 was compared with the validated Greek versions of PDQ-8 and EQ-5D-5L for criterion validity purposes.

The current study demonstrated that PDQoL7 is an acceptable construct for the determination of HRQoL in PD patients with content validity, convergent validity, internal consistency reliability and non-redundant items that reflect the effect of PD on various aspects of patients’ life.

At first, participants’ demographic and clinical characteristics were in agreement with epidemiological data on PD, revealing that the sample was representative of the PD population [16, 40, 56–59]. Moreover, although there was a strong correlation both between the PDQoL7 and PDQ-8 summary indices and within each of these tools with the same demographic and clinical parameters, greater variance was accounted for by PDQoL7 compared to PDQ-8. The parameters that contributed to variance in PDQoL7, namely wearing-off, LID and non-motor symptoms are strong predictors of PD progression that contribute to variance in HRQoL assessments in other studies as well [8, 60–69]. Τhere was no evidence of floor or ceiling effects for PDQoL7. The item to total correlations (range of 0.29–0.63) of PDQoL7 were also satisfactory. The degree to which repeated measurements in the same patients resulted in similar answers is usually investigated in HRQoL tools at 1–2 week intervals and was not investigated in the current study due to the 1-week recall period of PDQoL7 [54].

In addition, based on Cronbach’s a value, PDQoL7 had potentially greater internal consistency and reliability than PDQ-8. The expected strong negative correlation between the EQ-5D-5L index and PDQ-8 scores that has been reported in literature (r_s_: -0.60 up to -0.78) was observed between EQ-5D-5L and PDQoL7 scores as well [44]. The negative correlation is expected, as PDQoL7/PDQ-8 and EQ-5D-5L scores run in opposite directions. Nevertheless, the stronger negative correlation between EQ-5D-5L and PDQoL7 index scores compared to EQ-5D-5L and PDQ-8 indicates that PDQoL7 is potentially more comprehensive than PDQ-8.

The validity of grouping the items of PDQ-39 and its 8-item short form (PDQ-8) in 8 domains and the initially proposed unidimensionality of PDQ-39 have been questioned [70, 71]. The available evidence suggests, though not confirming, that PDQ-39 may be multidimensional with 3 HRQoL domains, namely physical-functioning, cognition, and socioemotional [11, 14–16, 70, 72]. PDQoL7 was also found to be multidimensional with 3 domains. Based on the PDQoL7 items that cluster on the same components, we may suggest that component 1 (“Mobility” and “Skills & Personal-care”) represents physical functioning, component 2 (“Social life/Communication” and “Emotional status) represents socioemotional status and component 3 (“Cognition/Sleep”) represents cognition, bearing in mind the correlation between sleep dysregulation and cognitive decline [32, 37, 63, 73]. The clustering of sleep domain or sleep disturbances under the cognition domain has been demonstrated in other studies, too [30, 63]. In the study by Kim et al. (2014) that investigated inter-relationships between non-motor symptoms (assessed via the Non-Motor Symptoms Scale), two types of non-motor symptom clusters were identified, with sleep/fatigue clustering together with mood, attention/memory, urinary and miscellaneous symptoms (cluster 1), whereas the other cluster included perceptual problems, gastrointestinal issues, and cardiovascular symptoms (cluster 2). Although the three-dimensional structure of PDQoL7 warrants confirmation on a larger sample of patients, nevertheless, it mirrors the suggested three-dimensional structure of PDQ-39, which is more comprehensive than PDQ-8, but also an extensive and time consuming tool [7, 9, 70, 74]. Moreover, compared to the two-dimensional structure of PDQ-8, the proposed three-dimensional structure of PDQoL7 complies with the International Classification of Functioning, Disability and Health by WHO that identifies the components of health that are impacted by diseases or other health conditions and is important for healthcare decision-making purposes [7, 75]. Additionally, the three-dimensional structure of PDQoL7 accounted for greater variance that the corresponding two-dimensional structure of PDQ-8. Although reliability, which can be defined as the homogeneity in a measurement, is distinct from validity, which is associated with the accuracy of a measurement, reliability is a prerequisite for validity [76, 77]. Due to the multidimensional structure of PDQoL7, omega coefficient was also determined, because it is considered more reliable than Cronbach α coefficient for non-unidimensional tools [76, 78–81]. Omega hierarchical analysis confirmed that PDQoL7 was at least comparable to and slightly more reliable than PDQ-8. Though there is no universally accepted guideline for acceptable or adequate levels of omega reliability for clinical decision making, omega total coefficients should meet the same standards as α coefficients, with high omega total values indicating highly reliable multidimensional constructs [81]. Similarly, omega hierarchical coefficients should exceed 0.50 at a minimum [81]. Due to the lack of published evidence on the dimensionality of PDQ-8, omega coefficients have not been calculated for PDQ-8. Overall, the high omega total value as well as the omega hierarchical > 0.5 for PDQoL7 add further evidence to PDQoL7 as a reliable three-dimensional structure tool.

The study has some limitations that have to be acknowledged to logically evaluate the results. The small sample size is a limitation that may also interfere with the reliability of structure detection. Nevertheless, the purpose of this study was to provide preliminary evidence on the comparative value of PDQoL7 versus PDQ-8. The cross-sectional design is another limitation as PD is a chronic disease and test–retest reliability was not determined. The responsiveness of PDQoL7 in longitudinal studies, the determination of meaningful change threshold and its evaluation on larger cohorts of PD patients across wider geographical regions will be investigated in subsequent studies. However, the consecutive patient enrollment of unselected patients that allowed for the inclusion of PD patients with various clinical and demographic characteristics recruited from a community-based sample reduces the potential for selection bias and may contribute to the low concordance in patients’ assessments and is one of the strengths of the current study. HRQoL assessments are subjective and reflect the demographic and clinical characteristics of the patients under study as well as their health state at the time of the assessments. The assessments and questionnaires were administered to all subjects concurrently under the same conditions and were, therefore, normalized for the patients’ health state and perception of health state at the time. Besides, PD is a heterogeneous disease both in terms of symptoms and progression, thus, the heterogeneity in the patients’ clinical characteristics is anticipated [82].

Nevertheless, a strength of our study is that the questionnaires PDQ-8 and EQ-5D-5L have been validated both in PD patients and in Greek language. Furthermore, the current study confirmed existing published data on PDQ-8 [12, 16, 18, 20, 21, 72, 83–85], thus adding to the credibility of the results for PDQoL7. Additionally, the conduct of the study by trained neurologists specialized in movement disorders encompasses an ascertainment of a high level of accuracy in the recognition and evaluation of the different clinical symptoms and signs of the disease. The current results confirmed existing published evidence on PDQ-8, thus, adding further evidence on the validity of the results on PDQoL7. In addition to addressing the gaps of PDQ-8 in non-motor symptom burden, an advantage of PDQoL7 compared to PDQ-8 is its short recall period of 1 week. Recall bias (or memory bias) is the extent to which memory impairment can affect the assessment of a target construct, namely HRQoL, as PD patients suffer from mild cognitive impairment even before PD is diagnosed [86, 87]. Since the ability to accurately remember and report HRQoL affects the reliability and validity of the corresponding instruments and the conclusions that can be drawn, the 1-month recall period of PDQ-8 (or PDQ-39) compared to the 1-week recall period of PDQoL7 increases the potential for patient recall bias. Although the optimal recall period for HRQoL assessments has not been determined and should reflect a balance between minimizing recall bias and maximizing the generalizability of the conclusions [88, 89], PDQoL7 may be better suited for HRQoL assessments in PD patients [10, 86, 90].

## Conclusion

This study demonstrates that PDQoL7 is an adequate and easy to use construct-valid tool for the determination of HRQoL in PD patients. PDQoL7 has higher convergent validity and internal consistency (based on Cronbach’s α and omega coefficient) and is more comprehensive compared to PDQ-8 based on its higher correlation with EQ-5D-5L index score. Another advantage of PDQoL7 is the coverage of a broader range of symptoms that impact on quality of life, at any stage of PD, than PDQ-8. Furthermore, the 3-dimensional structure of PDQoL7 is in line with the International Classification of Functioning, Disability and Health and resembles that of the more extensive and time-consuming PDQ-39. PDQoL7 has no floor/ceiling effects. Although the same factors contribute to variance in PDQ-8 and PDQoL7, nevertheless, greater variance is accounted for by PDQoL7. Additionally, PDQoL7 assessments are less prone to bias in assessments by memory deficits due its short recall period [86, 90]. These data warrant investigation in a larger sample of patients and suggest that PDQoL7 could be used to substitute PDQ-8 in HRQoL assessments in PD patients. PDQoL7 may be preferred in settings where a short, comprehensive and reliable HRQoL construct is required that can be regularly used for the objective assessment of motor and non-motor symptom burden across PD patients with various clinical characteristics.

## Supplementary Information

**Additional file 1**. Supplementary material that includes Figure 1 and Tables 1-6 can be found in the respective file submitted via the Manuscript Tracking System.

## Data Availability

The datasets used and/or analysed during the current study are available from the corresponding author on reasonable request.

## Abbreviations

AR: Akinetic-rigid
EQ-5D-5L: 5-Level EQ-5D
HR: Health-related
HRQoL: Health-related quality of life
MDS-UPDRS: Movement Disorder Society Unified Parkinson's Disease Rating Scale
LID: Levodopa-induced dyskinesia
NMSQuest: Non-motor symptoms questionnaire
NMSS: Non-motor symptoms scale
PD: Parkinson’s disease
PDQ-8: Parkinson's disease questionnaire-8 item version
PDQ-8^GrV^: Parkinson's disease questionnaire-8 item Greek validated version
PDQ-39: Parkinson’s disease questionnaire-39 item version
PDQL: Parkinson’s disease quality of life questionnaire
PIMS: Parkinson’s impact scale
PLQ: Parkinson LebensQualität questionnaire
QoL: Quality of life
r_s_: Spearman’s correlation
TD: Tremor-dominant
