# Supplementary material for: Evaluation and validation of a patient-reported quality-of-life questionnaire for Parkinson’s disease
Source: J Patient Rep Outcomes. 2022 Mar 2;6:17. doi: 10.1186/s41687-022-00427-0 (PMC8891413; doi:10.1186/s41687-022-00427-0)
Supplement: Supplementary file 1 — Additional file 1. Supplementary material that includes Figure 1 and Tables 1-6 can be found in the respective file submitted via the Manuscript Tracking System. [file 41687_2022_427_MOESM1_ESM.docx]

**Article title**

Evaluation and Validation of a Patient-Reported Quality-of-Life Questionnaire for Parkinson’s Disease

Supplementary Material

Figure 1: PDQoL7 & PDQ-8 questionnaires

**PDQoL7 Questionnaire**

**Please select one answer only** **for each question (a. Never, b. Rarely, c. Sometimes, d. Often, e. Always)**

**Please evaluate how often in the last week because of Parkinson's:**

**1. (Mobility)**

You had difficulty moving (e.g. difficulty walking, moving your hands or changing the bed position).

**2. (Skills & Personal Care)**

You had a problem with work, driving, hobbies, housework, personal hygiene, dressing etc.

**3. (Social Life-Communication)**

You felt isolated or had problems communicating with those around you (companion, family, friends, neighbors etc.)

**4. (Problems with non-motor symptoms)**

You had unpleasant symptoms such as pain, fatigue, numbness, dizziness while standing up, salivation, frequent urination, constipation.

**5. (Emotional status)**

You felt anxious, sad or depressed

**6. (Mental status)**

You have forgotten something important, lost your attention to something you were watching, or failed to put your thoughts in order.

**7. (Sleep)**

Do you have sleep problems or overnight symptoms which interrupt your sleep, or drowsiness during the day?

**PDQ-8 Questionnaire**

**Please select one answer only for each question (a. Never, b. Rarely, c. Sometimes, d. Often, e. Always).**

**Please evaluate how often in the last month because of Parkinson's ...:**

**1. (Mobility)**

Had difficulty getting around in public?

**2. (Activities of daily living)**

Had difficulty dressing yourself?

**3. (Emotional well-being)**

Felt depressed?

**4. (Social support)**

Had problems with your close personal relationships?

**5. (Cognition)**

Had problems with your concentration, e.g. when reading or watching TV?

**6. (Communication)**

Felt unable to communicate with people properly?

**7. (Bodily discomfort)**

Had painful muscle cramps or spasms?

**8. (Stigma)**

Felt embarrassed in public due to having Parkinson’s disease?

Table 1: Demographic and clinical characteristics

| **Characteristics** | ***60 patients*** |
| --- | --- |
| **Gender** |  |
| Male | 33 (55%) |
| Female | 27 (45%) |
| **Age** |  |
| Mean ± SD | 64.52 ± 9.39 |
| Age range | % of patients |
| <60 | 15 (25%) |
| 60-69 | 29 (48.4%) |
| 70-80 | 14 (23.3%) |
| >80 | 2 (3.3%) |
| Number of years since diagnosis (Mean ± SD) | 10.73±6.40 |
| **Modified Hoehn & Yahr stage, n (%)** | **% of patients** |
| Stage 1 | 12 (20%) |
| Stage 1.5 | 3 (5%) |
| Stage 2 | 15 (25%) |
| Stage 2.5 | 16 (26.7%) |
| Stage 3 | 13 (21.7%) |
| Stage 4 | 1 (1.7%) |
| **PD Subtype** | **% of patients** |
| **Motor signs^*^** |  |
| Akinetic-rigid | 30 (50%) |
| Tremor-dominant | 29 (48.3%) |
| Many autonomic symptoms | 2 (3.3%) |
| Not available | 1 (1.7%) |
| **Onset** |  |
| Early onset (< 50 years) | 18 (30%) |
| Expected onset (50-69 years) | 38 (63.3%) |
| Late onset (> 70 years) | 4 (6.7%) |
| **Cognitive Impairment** | **% of patients** |
| 0=No cognitive impairment | 27 (45%) |
| 1=Slight problems | 17 (28.3%) |
| 2=Mild problems | 14 (23.3%) |
| 3=Moderate problems | 2 (3.3%) |
| 4=Severe problems | 0 (0%) |
| **Motor fluctuations: Functional impact** |  |
| **Wearing-Off score (Mean ± SD)** | **26.58±18.03** |
| Wearing-Off | **% of patients** |
| 0=Normal | 10 (16.7%) |
| 1=Slight problems | 12 (20%) |
| 2=Mild problems | 19 (31.7%) |
| 3=Moderate problems | 19 (31.7%) |
| 4=Severe problems | 0 (0%) |
| **Motor fluctuations: Time spent in Off state** |  |
| % Off (Mean ± SD) | 16.15±14.14 |
| % Off | **% of patients** |
| 0=Normal (No off time) | 10 (16.7%) |
| 1=Slight (1-25% of walking day) | 25 (50%) |
| 2=Mild (26-50% of walking day) | 14 (23.3%) |
| 3=Moderate (51-75% of walking day) | 1 (1.7%) |
| 4=Severe (76-100% of walking day) | 0 (0%) |
| **Dyskinesias: Functional impact of LID** |  |
| LID score (Mean ± SD) | 14.83±14.50 |
| LID score | % of patients |
| 0=Normal | 25 (41.7%) |
| 1=Slight problems | 14 (23.3%) |
| 2=Mild problems | 12 (20%) |
| 3=Moderate problems | 8 (13.3%) |
| 4=Severe problems | 1 (1.7%) |
| **Dyskinesias: Time spent with LID** |  |
| % Dyskinesia-LID (Mean ± SD) | 7.52±9.57 |
| % Dyskinesia-LID | **% of patients** |
| 0=Normal (No dyskinesias) | 24 (40%) |
| 1=Slight (1-25% of walking day) | 33 (55%) |
| 2=Mild (26-50% of walking day) | 3 (5%) |
| 3=Moderate (51-75% of walking day) | 0 (0%) |
| 4=Severe (76-100% of walking day) | 0 (0%) |
| **NMS Quest** | **% of patients** |
| 1-10 symptoms | 26 (43.3%) |
| 11-20 symptoms | 21 (35%) |
| 21-30 symptoms | 13 (21.7%) |
| **Depressed Mood** | **% of patients** |
| 0=Normal | 3 (5%) |
| 1=Slight problems | 18 (30%) |
| 2=Mild problems | 26 (43.3%) |
| 3=Moderate problems | 13 (21.7%) |
| 4=Severe problems | 0 (0%) |
| **PDQOL7 total score** |  |
| **Mean ± SD** | **14.58±5.55** |
| **Median (IQR)** | **14.00 (10.00-19.00)** |
| **Min, Max** | **2.00, 24.00** |
| **PDQ-8 total score** |  |
| **Mean ± SD** | **11.82±6.04** |
| **Median (IQR)** | **12.00 (6.00-17.00)** |
| **Min, Max** | **0.00, 23.00** |
| **EQ-5D-5L** |  |
| **Mean ± SD** | 0.41**±** 0.27 |

*^*^There was 1 patient with both AR+AS and 1 patient with both TD+AS.*

Table 2: Summary of responses to PDQoL7 and PDQ-8 items

| **PDQoL7 items** | **Median** | **Quartile** | **Floor/Ceiling %** |
| --- | --- | --- | --- |
| **Mobility** | 3.0 | 2.0-4.0 | 3.3%/28.3% |
| **Skills & Personal care** | 3.0 | 1.0-3.0 | 11.7%/20% |
| **Social Life & Communication** | 1.5 | 0-2.0 | 35%/1.7% |
| **Problems from non-motor symptoms** | 3.0 | 2.0-3.0 | 3.3%/20% |
| **Emotional status** | 2.0 | 1.0-3.0 | 13.3%/8.3% |
| **Mental status** | 1.0 | 0-2.0 | 33.3%/0% |
| **Sleep** | 3.0 | 2.0-3.0 | 1.7%/11.7% |
| **PDQ-8 items** | **Median** | **Quartile** | **Floor/Ceiling %** |
| **Mobility** | 2.0 | 1.0-3.0 | 23.3%/20% |
| **Activities of daily living** | 2.0 | 1.0-3.0 | 21.7%/10% |
| **Emotional well-being** | 2.0 | 1.0-3.0 | 20%/5% |
| **Social support** | 1.0 | 0-2.0 | 36.7%/0% |
| **Cognitions** | 1.0 | 0-2.0 | 35%/1.7% |
| **Communication** | 1.0 | 0-2.0 | 35%/1.7% |
| **Bodily discomfort** | 1.0 | 0-2.0 | 28.3%/3.3% |
| **Stigma** | 2.0 | 0.3-2.0 | 25%/5% |

Table 3: Principal component factor analysis for PDQoL7

| **PDQ-7 items** | **Factor loadings^*^** | | |
| --- | --- | --- | --- |
|  | **Component 1** | **Component 2** | **Component 3** |
| **Mobility** | 0.903 | 0.166 | 0.031 |
| **Skills & Personal care** | 0.882 | 0.139 | 0.252 |
| **Social life & Communication** | 0.130 | 0.861 | -0.027 |
| **Problems from non-motor symptoms** | 0.567 | 0.591 | -0.144 |
| **Emotional state** | 0.118 | 0.804 | 0.251 |
| **Mental state** | 0.398 | 0.492 | 0.337 |
| **Sleep** | 0.107 | 0.088 | 0.951 |
| **% Variance explained** | 30.23% | 59.27% | 75.96% |
| **Component eigenvalue** | 2.12 | 2.03 | 1.17 |

********Principal component factor analysis (with rotation – Varimax method with Kaiser normalization)*

Table 4: Principal component factor analysis for PDQ8

| **PDQ-8 items** | **Factor loadings*** | |  |
| --- | --- | --- | --- |
|  | **Component 1** | **Component 2** | |
| **Mobility** | 0.743 | 0.351 | |
| **Activities of daily living** | 0.816 | 0.282 | |
| **Emotional well-being** | 0.488 | 0.438 | |
| **Social support** | 0.070 | 0.846 | |
| **Cognitions** | 0.688 | 0.246 | |
| **Communication** | 0.118 | 0.763 | |
| **Bodily discomfort** | 0.702 | -0.296 | |
| **Stigma** | 0.252 | 0.680 | |
| **% Variance explained** | 31.33% | 60.12% | |
| **Component eigenvalue** | 2.51 | 2.30 | |

******* *Principal component factor analysis (with rotation – Varimax method with Kaiser normalization*

Table 5: Hierarchical cluster analysis for PDQoL7 and PDQ-8 questionnaires

| **Clusters** | **PDQoL7** | **Clusters** | **PDQ-8** |
| --- | --- | --- | --- |
| **Cluster 1** | Mobility, Problems from non-motor symptoms, Skills & Personal care | **Cluster 1** | Social support, Communication, Stigma |
| **Cluster 2** | Sleep | **Cluster 2** | Cognitions |
|  |  | **Cluster 3** | Bodily discomfort |
| **Cluster 3** | Social life & Communication, Emotional state, Mental state | **Cluster 4** | Mobility, Activities of daily living |
|  |  | **Cluster 5** | Emotional well-being |

Table 6: Univariate regression analysis for PDQoL7 and PDQ-8 scores

| **Demographic/Disease characteristics** | **PDQoL7** | | | | **PDQ-8** | | | |
| --- | --- | --- | --- | --- | --- | --- | --- | --- |
|  | ***Adjusted R^2^*** | ***Estimate (95% CI)*** | ***Standard error*** | ***ANOVA test (F, p-value)*** | ***Adjusted R^2^*** | ***Estimate (95% CI)*** | ***Standard error*** | ***ANOVA test (F, p-value)*** |
| **Age** | 0.012 | 0.10 (-0.05, 0.25) | 0.77 | 1.72, 0.195 | 0.025 | 0.13 (-0.03, 0.30) | 0.08 | 2.52, 0.118 |
| **Number of years since diagnosis** | 0.266 | 0.458 (0.27, 0.65) | 0.09 | 22.43, <0.001* | 0.279 | 0.51 (0.30, 0.72) | 0.10 | 23.77, <0.001* |
| **Modified Hoehn & Yahr stage** | 0.303 | 4.22 (2.58, 5.85) | 0.82 | 26.70, <0.001* | 0.292 | 4.50 (2.72, 6.29) | 0.89 | 25.38, <0.001* |
| **PD Subtype** | -0.016 | -0.03 (-0.21, 0.16) | 0.09 | 0.07, 0.798 | -0.017 | 0.02 (-0.19, 0.22) | 0.10 | 0.03, 0.863 |
| Motor signs | -0.017 | 0.13 (-1.79, 2.05) | 0.96 | 0.02, 0.893 | -0.014 | -0.42 (-2.50, 1.67) | 1.04 | 0.16, 0.691 |
| PD onset | 0.004 | -1.43 (-4.00, 1.13) | 1.28 | 1.25, 0.268 | -0.008 | -1.04 (-3.85, 1.76) | 1.40 | 0.56, 0.459 |
| **Cognitive impairment** | 0.319 | 3.55 (2.22, 4.88) | 0.66 | 28.64, <0.001* | 0.313 | 3.83 (2.38, 4.88) | 0.73 | 27.89, <0.001* |
| **Disability-Off index** | 0.456 | 0.27 (0.19, 0.34) | 0.04 | 50.39, <0.001* | 0.438 | 0.29 (0.20, 0.37) | 0.04 | 46.98, <0.001* |
| **Disability-LID index** | 0.062 | 0.162 (0.02, 0.31) | 0.07 | 4.88, 0.031* | 0.096 | 0.21 (0.05, 0.37) | 0.08 | 7.28, 0.009* |
| **NMSQuest** | 0.454 | 4.83 (3.46, 6.19) | 0.68 | 50.05, <0.001* | 0.280 | 4.16 (2.46, 5.87) | 0.85 | 23.89, <0.001* |
| **Depressed mood** | 0.412 | 4.33 (2.99, 5.66) | 0.67 | 42.40, <0.001* | 0.340 | 4.29 (2.76, 5.83) | 0.77 | 31.46, <0.001* |

**Statistically significant factors. NMSQuest: Non-motor symptom questionnaire.*
